# Strength and Acid Resistance of Ceramic-Based Self-Compacting Alkali-Activated Concrete: Optimizing and Predicting Assessment

**DOI:** 10.3390/ma14206208

**Published:** 2021-10-19

**Authors:** Hassan Amer Algaifi, Mohammad Iqbal Khan, Shahiron Shahidan, Galal Fares, Yassir M. Abbas, Ghasan Fahim Huseien, Babatunde Abiodun Salami, Hisham Alabduljabbar

**Affiliations:** 1 Faculty of Civil Engineering and Built Environmental, Universiti Tun Hussein Onn Malaysia, Parit Raja 86400, Malaysia; shahiron@uthm.edu.my; 2 Department of Civil Engineering, College of Engineering, King Saud University, Riyadh 11421, Saudi Arabia; galfares@ksu.edu.sa (G.F.); yabbas@ksu.edu.sa (Y.M.A.); 3 Department of Building, School of Design and Environment, National University of Singapore, Singapore 117566, Singapore; 4 Interdisciplinary Research Center for Construction and Building Materials, King Fahd University of Petroleum and Minerals, Dhahran 31261, Saudi Arabia; salami@kfupm.edu.sa; 5 Department of Civil Engineering, College of Engineering, Prince Sattam bin Abdulaziz University, Alkharj 11942, Saudi Arabia; h.alabduljabbar@psau.edu.sa

**Keywords:** mathematical assessment, optimization, self-compacting alkali-activated concrete, granulated blast furnace slag, ceramic tile waste, durability, strength, microstructure

## Abstract

The development of self-compacting alkali-activated concrete (SCAAC) has become a hot topic in the scientific community; however, most of the existing literature focuses on the utilization of fly ash (FA), ground blast furnace slag (GBFS), silica fume (SF), and rice husk ash (RHA) as the binder. In this study, both the experimental and theoretical assessments using response surface methodology (RSM) were taken into account to optimize and predict the optimal content of ceramic waste powder (CWP) in GBFS-based self-compacting alkali-activated concrete, thus promoting the utilization of ceramic waste in construction engineering. Based on the suggested design array from the RSM model, experimental tests were first carried out to determine the optimum CWP content to achieve reasonable compressive, tensile, and flexural strengths in the SCAAC when exposed to ambient conditions, as well as to minimize its strength loss, weight loss, and UPVL upon exposure to acid attack. Based on the results, the optimum content of CWP that satisfied both the strength and durability aspects was 31%. In particular, a reasonable reduction in the compressive strength of 16% was recorded compared to that of the control specimen (without ceramic). Meanwhile, the compressive strength loss of SCAAC when exposed to acid attack minimized to 59.17%, which was lower than that of the control specimen (74.2%). Furthermore, the developed RSM models were found to be reliable and accurate, with minimum errors (RMSE < 1.337). In addition, a strong correlation (*R* > 0.99, *R*^2^ < 0.99, adj. *R*^2^ < 0.98) was observed between the predicted and actual data. Moreover, the significance of the models was also proven via ANOVA, in which *p*-values of less than 0.001 and high F-values were recorded for all equations.

## 1. Introduction

Owing to the rapid development in the construction industry, concerns about air pollution and industrial waste material have increased. As such, researchers have shifted their attention to developing green and sustainable concrete. For example, ceramic waste was utilized either as a partial replacement of aggregates [1] or cement [2] in a concrete matrix. It was found that CWP was able to produce green concrete with improved mechanical properties [3].

On the other hand, alkali-activated concrete (AAC) has emerged as one of the top trending concrete alternatives, particularly within the scientific community, owing to environmental considerations [4,5,6,7]. Free-cement-based alkali-activated concrete was developed using waste binders such as silica fume (SF), granulated blast furnace slag (GBFS), fly ash (FA), and palm oil fuel ash (POFA) [8,9,10,11]. The efficiency of the developed AAC was experimentally investigated and assessed based on the outcome of the mechanical properties and durability [12,13,14]. However, such classical and traditional methods require a large number of experiments to obtain the optimum content of the involved chemical reaction parameters. In addition to the variation in the experimental tests, the variable interaction investigation is also challenging via experimentation alone. Consequently, in recent years, many researchers have shifted their attention to adopting an optimization and predictive technique prior to conducting experimental studies [15,16]. For example, He et al. [17] used a simplex-centroid design method to optimize and assess the effects of ternary binders, including calcium aluminate cement (CAC), GBFS, and soda lime glass powder (GP), on the properties of alkali-activated mortar. The results of their study revealed that the optimum CAC content was higher than 10%, while the GBFS content should be lower than 5%. In addition, the GP content ranged from 77% to 90%. Recently, Dave et al. [18] successively optimised the mix of the proportions of the alkali-activated composite in an appropriate and systematic way using the Taguchi method. In another study by Kočí et al. [19], an alkali-activated aluminosilicate composition involving ceramic powder, water, sodium silicate, and siliceous sand was also optimised to effectively and quickly maximize the compressive strength using the downhill simplex method. Next, the partial replacement of FA by GBFS in AAC was theoretically evaluated using the Taguchi method in a study by Mehta et al. [20]. Based on their findings, the compressive strength of FA-based ACC increased with an increase in the GBFS content, owing to further polymerization products and the available calcium-based hydration product. In addition to the Taguchi method, the optimum content of ladle furnace slag in FA-based alkali-activated cement was obtained and assessed using another optimization method, namely response surface methodology (RSM) in a study by Pinheiro et al. [21]. The effect of waste glass powder on the flexural and compressive strengths of the alkali-activated material was also investigated using the RSM model [22].

In addition, Cong et al. [23] employed both experimental works and statistical approaches to investigate several properties of GBFS/FA-based alkali-activated concrete, including the modulus of elasticity, dynamic response under impact loads, direct tensile stress-strain relationship, and compressive stress-strain relationship. The predicted results revealed that the fly ash-based AAC-containing slag exhibited superior elasticity and ductility stress. In a previous study, a random forest (RF) approach was utilized by Gomaa et al. [24] to predict the fresh and hardened properties of fly ash-based alkali-activated concrete cured in an oven under ambient and moist conditions. The obtained results showed that the RF model was able to generate predictions with a higher accuracy than that of the experimental works. In the same context, the compressive strength of slag-based geopolymer concrete incorporating silica fume (SF) and natural zeolite (NZ) was estimated using a gene expression programming (GEP) model. The accuracy of the GEP model in predicting the compressive strength of GGBS-based geopolymer concrete under different amounts of SF, NZ, time, GBFS, and sodium hydroxide solution [25]. In particular, the respective values of *R*^2^ for both the training and validation phases were 0.918 and 0.94, respectively, indicating that both the experimental and predicted results were considered close and satisfactory. Other predictive and optimization methods are also available in the literature [26,27].

In the present study, both an experimental and a mathematical assessment using the RSM were taken into account to optimize and predict the optimal content of ceramic waste in GBFS-based self-compacting alkali-activated concrete. This is because the utilization of ceramic waste in AAC is still in its infancy [28,29]. The aim of this study was to provide in-depth information to promote the use of ceramic waste in the construction industry. To implement the aims of this study, experimental tests were first conducted based on the suggested design array of the central composite design (CCD). The obtained results were analyzed and assessed using the RSM models. Furthermore, ANOVA and error-statistics validation methods, such as *p*-value, F-value, and RMSE, were taken into account to evaluate the accuracy and sensitivity of the models. Moreover, the correlation statistics indicators, such as *R*^2^, adjusted *R*^2^, predicted *R*^2^, and *R*, were also computed to assess the fitness between the actual and predicted data. Finally, several microstructure tests, that is, FE-SEM, XDR, and XRF, were conducted to support the present findings.

## 2. Experimental and Mathematical Works

### 2.1. Design of Experiment Using Response Surface Methodology

Response surface methodology, specifically a face-centered central composite design (FC-CCD), was considered in the design experiments through five sequential steps. In the first step, the number of experiments was suggested and determined using the FC-CCD. In the second step, the experimental results were collected and analyzed using Design-Expert software (Version 12, Minneapolis MN 55413, U.S.). A numerical model was constructed using a second-order polynomial equation. Subsequently, the accuracy and performance of the models were verified using analysis of variance (ANOVA). Finally, the optimum CWP/GBFS ratio was obtained using the desirability function.

**Figure 1:**
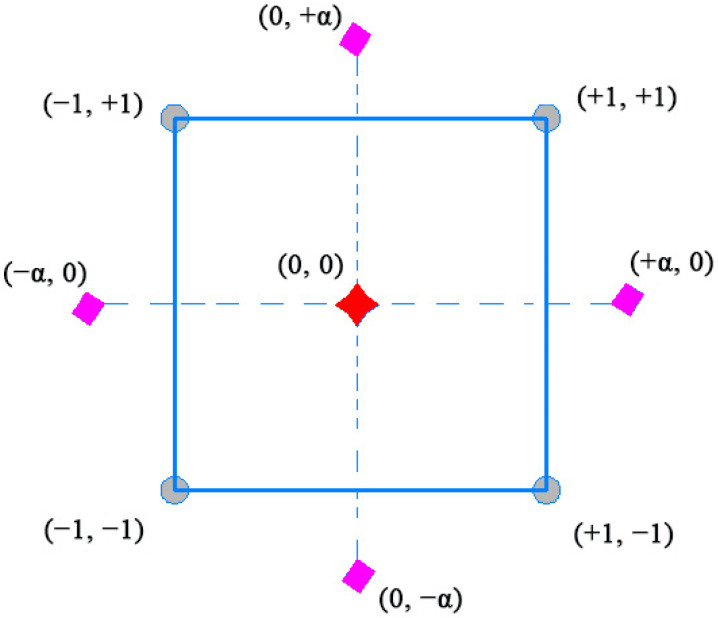
Schematic representation of FC-CCD.

Two independent variables and two levels were used in this study. The GBFS replaced by the CWP was the first independent variable (*d*_0_), whereas time (*d*_1_) was the second. As shown in Figure 1, FC-CCD involves three types of points, namely factorial points, axial points, and central points, according to their locations. For example, the square vertices point with a coded value of −1 and +1 are related to the factorial point. Moreover, the points situated at the centre of each face and far from the centre of the square with a distance of ±α referred to the axial or star points, while the central point situated in the centre of the square had a value of zero.

Consequently, the suggested number of experimental tests was 13 according to Equation (1), where *Z*_0_ and *Z* are the real values of the independent variables at the centre point and the real value of the independent value, respectively, and α and *R* are the step change value and the coded value of the independent variable, respectively. It should be noted that the main goal of using five central points was to evaluate the pure error in the proposed models. Similarly, the relationship between the coded values and the actual values of the experimental tests is expressed using Equation (2), where *m* is the number of centre points. Moreover, Equation (3) represents the general form of the second-order polynomial equation which was used to develop the model, where *β_i_* denotes the linear coefficients, *β*_0_ corresponds to the intercept of the model, and *β_ii_* denotes the quadratic coefficients.

(1) $$Q=2^{n}+2n+m$$

(2) $$R=\frac{Z-Z_{0}}{\alpha }$$

(3) $$Y=\beta_{0}+\sum_{i}^{k}\beta_{i}d_{i}+\sum_{i}^{k}\beta_{ii}d_{i}^{2}+\sum_{ij}^{k}\beta_{ij}d_{ij}\;$$

Herein, two RSM models, namely Model 1 and Model 2, were developed, as shown in Table 1. The compressive strength (*CS*), tensile strength (*TS*), and flexural strength (*FS*) of the GBFS-based SCACC containing CWP exposed to ambient conditions was evaluated in the first model at time intervals of 3 to 56 days. The second model was developed to determine the optimal strength loss (*SL*), weight loss (*WL*), and ultrasonic pulse velocity (*UPVL*) of the acid-treated SCACC at time intervals of 90 to 360 days.

In the same context, the accuracy of the developed equations was verified using an ANOVA. ANOVA is an integrated mathematical and statistical tool used to verify the significance and strength of a proposed model using several statistical validation indicators, such as *p*-value, F-value, adequate precision, *R*^2^, predicted *R*^2^, and adjusted *R*^2^. In particular, the developed equation could be classified as significant if the *p*-value was less than 0.005 and the F-value was high (>1). Similarly, the value of the adequate precision should be greater than four to demonstrate that the model has less error prediction. Moreover, a strong correlation between the actual and estimated results could be obtained if *R*^2^ was greater than 0.7. In addition, the model could be used for further prediction when the differences between the predicted *R*^2^ and adjusted *R*^2^ were less than 0.2.

To determine and assess the statistical validation indicators, several parameters should be first calculated beforehand, such as the total sum of squares (*SS_T_*), degree of freedom (*DF*), and residual sum of square (*SS_R_*). Both the *SS_R_* and *SS_T_* were obtained using Equations (4) and (5), respectively, where *Y_A_* and *Y_P_* denote the actual and predicted values, respectively, and $\bar{Y_{A}}$ is the average actual value. Consequently, *R*^2^ was determined using Equation (6).

(4) $$SS_{R}={\sum_{i=1}^{n}\left(Y_{A}-Y_{P}\right)}^{2}$$

(5) $$SS_{T}={\sum_{i=1}^{n}\left({\bar{Y}}_{A}-Y_{A}\right)}^{2}$$

(6) $$\begin{array}{ll} R^{2} & =1-\frac{SS_{R}}{SS_{T}}\\ & =1-\frac{{\sum_{i=1}^{n}\left(Y_{A}-Y_{P}\right)}^{2}}{{\sum_{i=1}^{n}\left({\bar{Y}}_{A}-Y_{A}\right)}^{2}} \end{array}$$

Similarly, the degree of freedom for the variance of dependent variables (*DF_T_*) and the degree of freedom for the residual error (*DF_R_*) were calculated to obtain the adjusted *R*^2^ using Equation (7). Meanwhile, the predicted *R*^2^ was determined using Equation (8), where *W* refers to the estimated residual sum of squares without the *i_th_*. In addition, Equation (9) represents the adequate precision (*SN*), which is also essential for assessing the signal-to-noise ratio, where σ^2^ refers to the residual mean square and *p* is the number of terms in the model.

(7) $$R_{adj}^{2}=1-\frac{SS_{R}/DF_{R}}{SS_{T}/DF_{T}}$$

(8) $$R_{pred}^{2}=1-\frac{W}{SS_{T}}$$

(9) $$SN=\frac{\max(Y_{p})-\min(Y_{p})}{\sqrt{\frac{p\sigma^{2}}{n}}}$$

### 2.2. Material and Mixing Process

In the present experimental work, two types of waste materials were adopted in addition to the alkali-activated binder, GBFS and CWP. The GBFS was collected from a local iron industry (Ipoh, Malaysia) and used as the main source of calcium and silica oxides in the preparation of the SCAAC specimens without applying any laboratory treatment. It is well known that GBFS exhibits cementing and pozzolanic properties and reacts hydraulically with water. As for its physical properties, the colour of GBFS is off-white. The medium particle size was estimated to be 12.8 µm from a particle-size analysis. From the Brunauer, Emmett and Teller (BET) test, the specific surface area and specific gravity of the GBFS specimen were found to be 13.6 m^2^/g and 2.89, respectively. From the viewpoint of chemistry, the chemical composition of both the CWP and the GBFS was determined using X-ray fluorescence (XRF) spectroscopy. The main composition of GBFS was calcium oxide (51.8% weight), followed by silica and aluminium (41.7 %). This result is in line with several other research results which reported that the synthesis of an alkali-activated paste is greatly dependent on the concentrations of CaO, SiO_2_, and Al_2_O_3_ oxides. These compounds are involved in the development of the N,C-(A)-S-H gels during geopolymerization.

Ceramic waste was collected from a tile ceramic waste industry (Johor, Malaysia). The ceramic waste was primarily processed using laboratory treatment prior to SCAAC specimen preparation. At an earlier stage, the ceramic waste was crushed using a crushing machine and sieved through a sieve with a mesh size of 600 µm. Subsequently, the obtained ceramic was ground for 6 h to produce a fine CWP of light grey colour with a particle size of 35 µm, a specific surface area of 12.2 m^2^/g, and a specific gravity of 2.61. Based on the chemical composition analysis, the silica and aluminium contents in the CWP were approximately 84.8% of the total element weight. Unlike GBFS, CWP presented a very low calcium oxide content. Moreover, neither CWP nor GBFS possessed a large amount of potassium oxide (K_2_O). On the other hand, CWP contained 13.5% sodium oxide (Na_2_O). Although this goes beyond the focus of the present study, there is evidence that the activation of the alkaline and geopolymerization depends significantly on the Na_2_O and K_2_O contents. It is also interesting to note that the CWP and GBFS conformed to ASTM C618, in which both materials exhibited extremely low loss of ignition values.

For the alkaline-activator solution preparation, two types of solutions were prepared and mixed: sodium silicate (NS) and sodium hydroxide (NH). Both NH and NS were supplied by QREC (Selangor, Malaysia). From the viewpoint of the analytical chemical grade, the NH exhibited a purity of 98%, while the NS solution contained Na_2_O (14.70 wt.%), SiO_2_ (29.5 wt.%), and H_2_O (55.80 wt.%). The molarity of the NaOH solution was maintained at 2M during the preparation of the NH solution. Following this, the solution was left to cool for 24 h prior to the addition of sodium silicate solution to prepare the final alkaline solution with a modulus solution ratio of 1.2 (SiO_2_/Na_2_O). The ratio of sodium silicate to sodium hydroxide (NS:NH) was maintained at 0.75 for all mixtures. In addition, both the local river sand, with a specific gravity of 2.62, and crushed granite were utilized as fine and coarse aggregates, respectively, to prepare the SCAAC specimens. The measured specific gravity and water absorption of the crushed granite were found to be 2.67 and 0.51%, respectively. To sustain the ratio of the alkaline solution to the binder (S:B), the supplied river sand was used under a saturated surface dry condition for all mixes.

For the design of the experiments, the SCAAC mixtures were coded and represented as shown in Table 1. It should be noted that the ratios of solution to binder (S:B) and sodium silicate to sodium hydroxide (NS:NH), the sodium hydroxide molarity, the solution modulus (SiO_2_:Na_2_O), and the content of the binder, river, and crushed granite were fixed at 0.50, 0.75, 2 M, 1.2, 484 kg/m^3^, 844 kg/m^3^, and 756 kg/m^3^, respectively, for all of the self-compacting alkali-activated mixtures. Several steps were taken into account when preparing the SCAAC specimen. First, both the CWP and GBFS were mixed and blended in a concrete mixer for 3 min. In this step, a dry condition was considered to ensure a uniform mixture between the coarse and fine aggregates. Subsequently, the alkaline solution was added to the mixture for 6 min at moderate speed. Several techniques were used to evaluate the required initial properties of the SCAAC mixtures, such as the filling and penetration capabilities, as well as the segregation resistance.

### 2.3. Test Methods

The European guidelines for self-compacting concrete, EFNARC (European Federation for Specialist Construction Chemicals and Concrete Systems) were adopted in this study to evaluate the fresh performance of the SCAAC mixtures. It was found that the inclusion of CWP as a GBFS replacement from 0% to 10%, 45%, and 80% led to an increase in the slum flow value from 560 mm to 605, 640, and 750 mm, respectively, while the T50 test showed an enhanced flow of fresh concrete and a time reduction from 6 s to 5.2, 5, and 3 s, respectively. An L-box test improved the workability in which the H2 to H1 ratio increased from 0.78 to 0.95.

According to ASTM C109, ASTM C496, and ASTM C78, the compressive strength (*CS*), splitting tensile (*TS*), and flexural strength (*FS*) tests were conducted at time intervals of 3, 7, 28, and 56 days. Samples of cubic (100 × 100 × 100) mm, cylindrical (200 × 100) mm, and prism (100 × 100 × 500) mm dimensions were fabricated for all the *CS, TS,* and *FS* tests, respectively. The average values from all the tests on all three specimen types were collected and compared to those of the control specimen prepared without any CWP content.

The durability and life service of the proposed concrete were measured in an aggressive environment by exposing the SCAAC specimen to a sulphuric acid solution. Deionized water was used to prepare the sulphuric acid (initial concentration is 96%) solution (H_2_SO_4_) at a concentration of 10%. The cured specimen at day 28 was evaluated with regard to the *CS*, weight, and ultrasonic pulse velocity (*UPVL*) before further immersion in the solution. To maintain the pH of the solution, a fresh H_2_SO_4_ solution was replaced every three months throughout the experiment. After 90, 180, and 360 days from the immersion date, the concrete specimen was examined, and several indicators were investigated for the performance assessment, such as compressive strength loss (*SL*), weight loss (*WL*), and *UPVL* according to the ASTM C267 specifications [30], as well as the surface and edge deterioration.

### 2.4. Microstructure

In this study, three microstructure tests were considered to evaluate the effect of the CWP content on the proposed concrete performance, including X-ray diffraction (XRD), Fourier transform infrared (FTIR) spectroscopy, and scanning electron microscopy (SEM). To analyse the obtained results, the XRD data were connected to Jade software (CA, USA), where inspection of the disordered stage of the alkali-activated pastes was enabled and carried out in the 2θ range of 5–90° at a step size of 0.02° and a 0.5 s/step scanning speed. The procedure involved positioning the sample on a brass stub holder fixed with carbon tape, prior to exposure to infrared radiation for five minutes for drying purposes. The sample was subsequently covered with a layer of gold using a blazer-sputtering coater. Patterns were documented at 20 kV and 1000× magnification. The vibration modes of the underlying chemical structures of the alkali-activated pastes were determined using an FTIR spectrophotometer. To assess the surface morphology of the tested alkali-activated pastes, the samples were coated in advance using a gold sputtering coater machine and tested using the SEM instrument by applying a similar magnification to the images.

## 3. Results and Discussion

### 3.1. Predicted Equations for Mechanical Properties and their Validation

Based on the present findings, six predictive quadratic equations were developed. In particular, model 1 reflects the mechanical properties of SCAAC exposed to ambient conditions, including compressive, flexural, and tensile strengths. The second model represents the behaviors of the SCAAC upon exposure to an acid attack, including compressive strength loss, weight loss, and UPVL. Indeed, these equations are important because the relationship between the involved parameters and responses can be quickly predicted and evaluated. The accuracy of these equations was evaluated using several statistical validation parameters, as listed in Table 2. For instance, the correlation between the predicted and actual data was assessed using the *R*^2^. It can be seen that the *R*^2^ values of the compressive, tensile, and flexural strengths were higher than 0.99. Similarly, the *R*^2^ values of compressive strength loss, weight loss, and UPVL were greater than 0.99. It was also found that the differences between the adj. *R*^2^ and predicted *R*^2^ values were lower than 0.056 for all data sets. Such results are considered an indicator of a strong correlation between the actual and estimated data. This is in line with Mohammed et al. [31], who stated that a model could be considered accurate and reliable if the differences between the adjusted and predicted *R*^2^ were lower than 0.2. In the same regard, a good agreement and fitness between the actual and estimated results was also proven, in which the ratio between *R*^2^ and adj. *R*^2^ was found to be high. This fact is consistent with Mohammed et al. [32], who demonstrated that the best fitness could be achieved if the *R*^2^ to adj. *R*^2^ ratio was high. In addition, the *R* values of the mechanical properties of SCAAC were higher than 0.995. Similarly, the *R* values of the strength loss, weight loss, and UPVL were 0.998, 0.9988, and 0.9999, respectively. As such, the developed models could be utilized for the purpose of prediction, which is in good agreement with previous studies. For example, Khan et al. [15] highlighted that the correlation between the predicted and the actual results could be considered as strong if the *R* value ranges were between 1 and 0.8, whereas moderate correlation could be achieved when the *R* value was greater than 0.5, but less than 0.8. This was also confirmed by Carrillo et al. [33]. In the same context, it can be seen that the value of adeq. precision was greater than four, indicating a perfect fit of the proposed models. Moreover, the adequacy and efficiency of the proposed models were verified using an error statistical indicator, namely RMSE. The implementation of statistical error indicators is necessary to assess the prediction error. It was observed that the RMSE values were less than 1.337 for all of the datasets, indicating high accuracy.

For the same considerations, ANOVA was used to investigate the applicability of the models using the *p*-value and F-value, as shown in Table 3. The *p*-value is considered to be one of the best mathematical methods for verifying the significance of a regression coefficient. When the *p*-value was smaller than 0.005, the model was significant. In contrast, an insignificant model was obtained when the *p*-value was greater than 0.005. The *p*-values of all models were found to be less than 0.0001, confirming that the models were significant. Similarly, the F-value was also used to assess the significance of the mean value variance. As is well known, a model can be considered significant if the F-value is higher, whereas a lower value indicates an insignificant model. In the developed models, it can be inferred that all the models were reliable and applicable for prediction owing to the high F-values. This inference is in good agreement with Ray et al. [34], who evaluated the predictive models of the compressive and splitting tensile strengths of concrete incorporating fine glass aggregate and condensed milk can fibers using F-values. Based on their findings, their models were considered accurate and significant owing to their high F-values.

**Figure 2:**
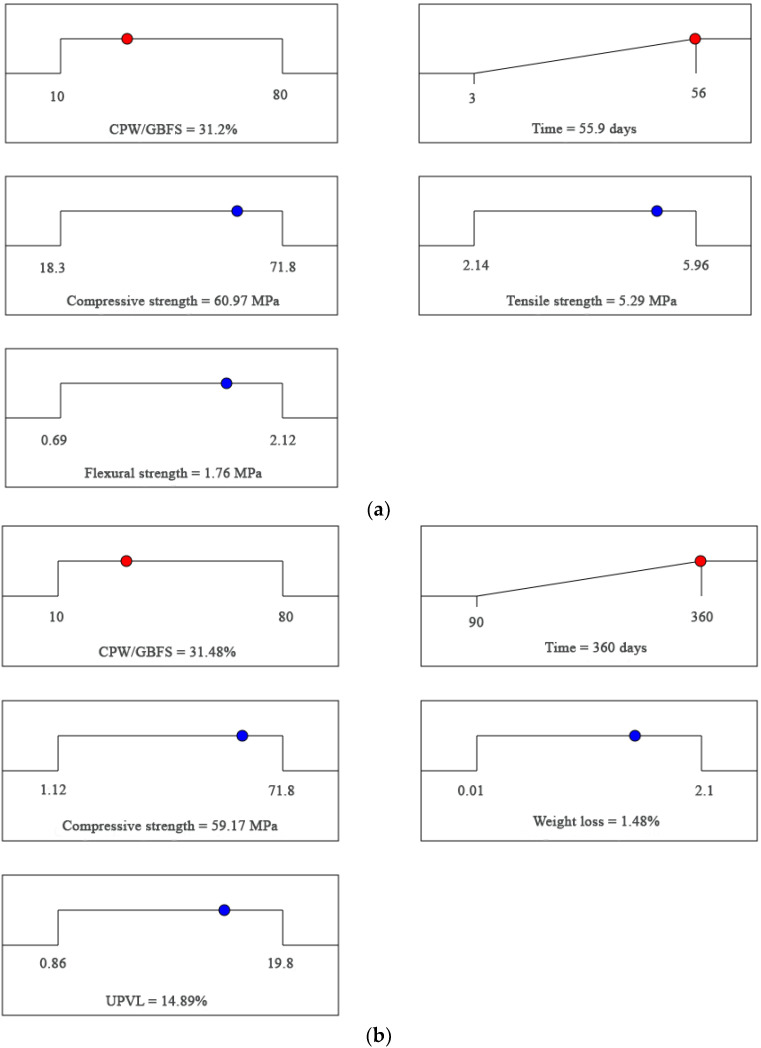
Optimum replacement percentage of GBFS with CWP in the aspects of (**a**) mechanical properties and (**b**) durability.

After verifying the developed equations, the optimum replacement percentage of GBFS by CWP was investigated using multi-objective optimization based on a desirability function. Indeed, the desirability function (*DR*) has been widely used owing to its proven ability to determine the optimal content that either maximizes or minimizes the target output. Equation (10) describes the desirability function, where *n* refers to the number of independent and dependent variables. Two independent variables, namely time and the CWP/GBFS ratio, were considered; however, the focus of the current study is more focused on the latter (the CWP/GBFS ratio). Therefore, two scenarios were considered to determine the optimum percentage of CWP. First, the optimum percentage of CWP was obtained on the basis of the maximum compressive, tensile, and flexural strengths achieved, whereas the second scenario aimed at obtaining the optimal content with a minimum strength loss. In general, it was found that the mechanical strength decreased with increasing ceramic content. In contrast, the durability properties, such as the weight and strength loss, as well as the UPVL, of the SCAAC exposed to acid attack improved. Based on Figure 2a,b, the optimum value of the CWP/GBFS ratio that satisfied both the strength and durability was 31%. This is because, in terms of mechanical properties, the decrease in compressive strength was 16% compared to that of the control specimen (without ceramic), which is considered an acceptable result. From the viewpoint of durability, the strength loss minimized to 59.17%, which is lower than that of the control specimen (74.2%). In other words, both the mechanical properties and the durability of the SCAAC could be considered acceptable when the replacement of the GBFS by the CWP was lower than 31%. Beyond this value, despite the fact that the durability properties of the SCAAC exposed to acid attack increased with an increase in the CWP content, a significant reduction in the compressive, tensile, and flexural strength was recorded.

(10) $$DR={\left(d_{1}\times d_{2}\times d_{3}\times \;\ldots \ldots d_{n}\right)}^{\left(1/n\right)}$$

**Figure 3:**
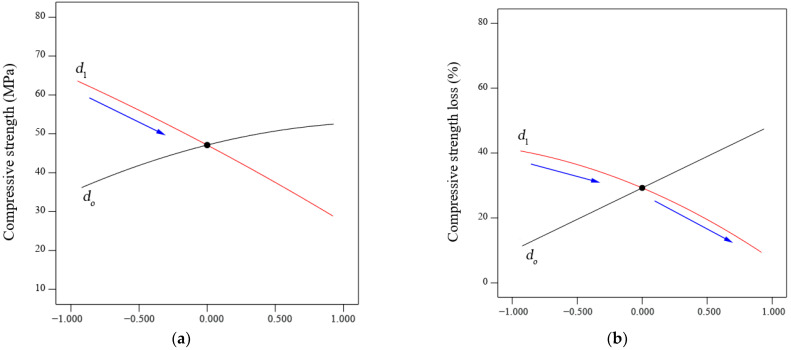
Perturbation curves for the (**a**) compressive strength of SCAAC (Model 1) and (**b**) strength loss of SCAAC when exposed to acid attack (Model 2).

On the other hand, the influence of the CWP:GBFS ratio content on the mechanical properties and durability of SCAAC was clearly observed. As illustrated in Figure 3a, a sharp slope is observed, indicating a significant impact. This fact is consistent with the ANOVA results in which the *p*-values of compressive, tensile, and flexural strength were less than 0.0001 and the F-value was high, confirming that the addition of ceramic adversely affected the mechanical properties of the SCAAC. In other words, the mechanical properties of SCAAC decreased with increasing CWP content.

In contrast, the strength loss, weight loss, and UPVL improved with an increase in CWP content. This fact can be observed in Figure 3b, in which the slope gradient for the ceramic content (*d*_1_) was high, confirming that the addition of CWP was also significant in terms of durability. These results were also confirmed by ANOVA. According to Tian et al. [35], the significance and sensitivity of a linear variable were proven with a *p*-value of less than 0.5. In the present study, the *p*-values for strength loss, weight loss, and UPVL were also less than 0.0001a and the F-value was high, indicating a significant interaction of CWP with respect to durability.

### 3.2. Effect of CWP Incorporation on the Mechanical Properties of AAC

**Figure 4:**
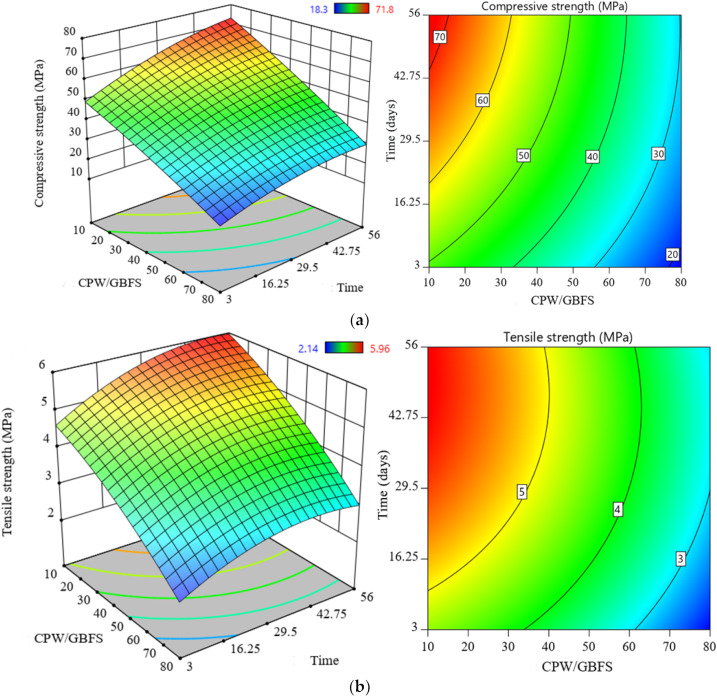

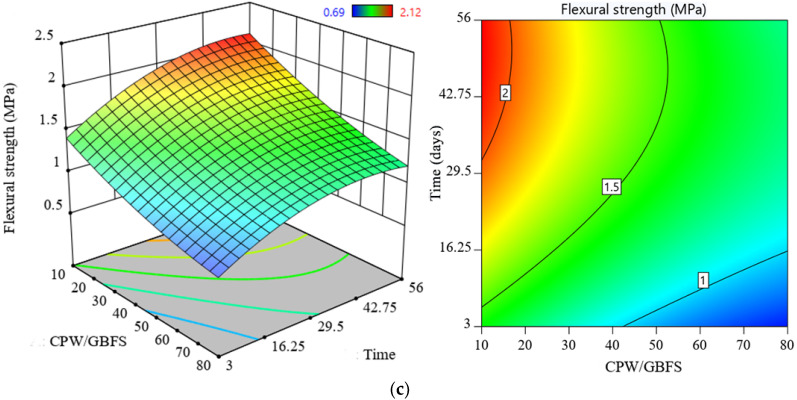
Strength evolution in the proposed concrete: (**a**) CS, (**b**) TS, and (**c**) FS.

Figure 4 illustrates the effects of the CWP incorporation as the GBFS replacement and curing age on the evolution of the compressive strength, tensile strength, and flexural strength of the SCAAC. As shown in Figure 4a, the inclusion of CWP in the alkali-activated matrix as a part of the binder negatively affected the compressive strength of the SCAAC exposed to ambient conditions. In contrast, the results showed that the development of CS monotonically improved with the increase in age. In general, at both the early and later ages, the compressive strength was highly influenced by the CWP content. At 28 days of curing age, the results showed that the increasing of the CWP content from 0% to 80% led to the compressive strength reduction from 70.1 MPa to 26.2 MPa, respectively.

The results of the splitting tensile strength of the SCAAC specimen prepared with various levels of GBFS replacement with CWP are also presented in Figure 4b. The results revealed a decreasing trend in tensile strength with increasing CWP content. However, all the proposed concrete specimens demonstrated an increase in strength development with increasing curing age. The highest splitting tensile strength value of 5.96 MPa was achieved by the control sample, and this value dropped to 2.93 MPa with an increase in the level of CWP to 80%. For flexural strength, a similar trend was observed, and the value of the strength decreased with an increase in the CWP content from 0% to 80% as the GBFS replacement. For example, with the increasing of the content of the CWP from 0 to 80%, the value of the flexural strength dramatically dropped from 2.2 to 1.19 MPa, respectively.

In general, the decrease in the strength of the proposed alkali-activated concrete incorporating ceramic is due to the limited amount of CaO present in the mix, in which the ratio of SiO_2_ to CaO is not balanced but increases steadily. A high content of CWP but a low content of CaO could negatively affect the chemical reaction rate and thus the productivity of C-(A)-S-H. In other words, the amount of C-(A)-S-H and the concrete strength are linearly dependent on the CaO content. This is consistent with a study by Rashad [36]. According to their study, the alkali-activated concrete strength decreased with increasing aluminosilicate content in materials such as fly ash. In the present study, the decrease in the CWP/GBFS-based alkali-activated concrete strength may be attributed to several reasons. For example, the chemical compositions of CWP and GBFS play a significant role in adversely affecting the alkali activation process of the binder. In addition, the decrease in the reaction rate of the CWP is considered as the second reason for the strength reduction [37]. Third, a high amount of CWP content could also lead to lower compactness and density of concrete, and thus, reduced strength. In the same context, the low sodium hydroxide concentration (2 M) is another reason for countering the lack of CaO content in the present study. Thus, it can be inferred that the compressive strength development in the SCAAC could only increase with the formation of the C-S-H and C-A-S-H gels as well as the N-A-S-H [29,38].

**Figure 5:**
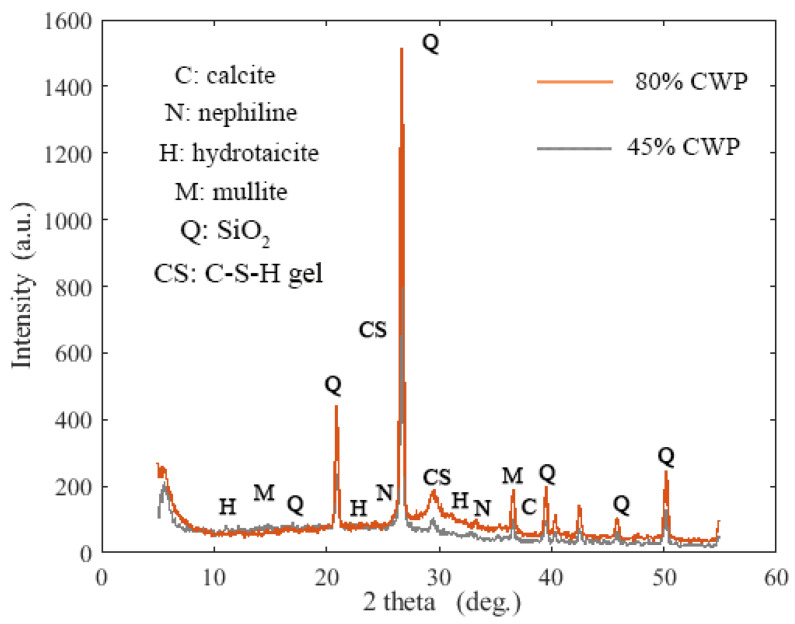
XRD results for the effect of GBFS replacement with CWP in SCAACs.

In the same regard, X-ray diffraction (XRD) analysis was also taken into account to analyse and support the results of the proposed SCAAC containing a high amount of CWP (45% and 80%). As shown in Figure 5, the hydrotalcite (Mg_6_Al_2_CO_3_OH_16_.4H_2_O) phase emerged at a peak around 10° [39]. In addition, a reduction in the C-S-H peak intensity was observed at 30° with increasing CWP concentration. The C-S-H is most probably substituted by hydrotalcite at the peak 43° with the replacement percentage of GBFS by CWP of 80%. Similarly, calcite was found to be substituted by quartz at peaks at 17°, 21°, 28°, 38.5°, and 51° when the amount of CWP was higher than 45%. It is also remarkable to note that increasing the CWP level leads to a decreased C-S-H product and an increased non-reacted silicate quantity, which confirmed the previous results where the concrete strength dropped from 70.1 to 26.2 MPa after 28 days. This is also in line with the previous literature [40], in which the productivity of the C-S-H gel and the calcite formulation could be diminished owing to the low CaO content. Moreover, the formation of C-S-H gel, N-A-S-H gel, and calcite was also detected [41].

**Figure 6:**
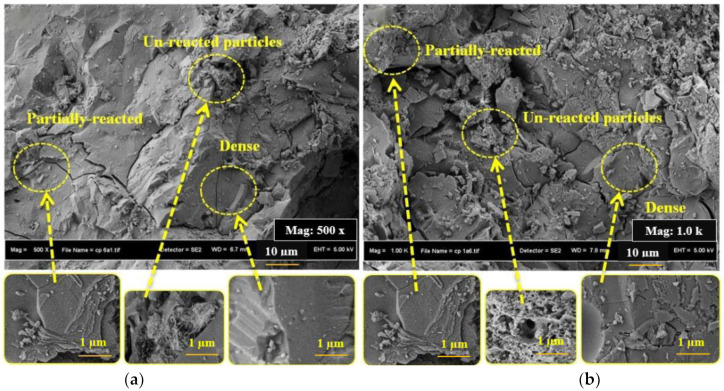
Surface morphology of SCAACs containing CWP at (**a**) 45% and (**b**) 80%.

On the other hand, the surface morphology of the SCAACs incorporating different replacement percentages of GBFS with CWP was also examined and analysed after 28 days using SEM analysis to support the present finding. In particular, concrete samples with replacement percentages of 45% and 80% were considered. Based on Figure 6a, the concrete specimen containing 45% CWP was found to be dense, and the quantity of both the partial reaction and the unreacted particles was small. The surface of the second concrete specimen (80% CWP) exhibited a large amount of partly reacted and unreacted particles, as shown in Figure 6b. As such, it can be inferred that poor morphology, a high degree of porosity, and a high quantity of unreacted quartz (SiO_2_) were clearly observed when the value of the CWP increased from 45% to 80%. In other words, a reduction in the GBFS and a high amount of CWP had a negative effect on the C-(A)-S-H gel formulation. In addition, it leads to the generation of non-reacted particles, including quartz, and a more partially reacted gel such as mullite [42].

**Figure 7:**
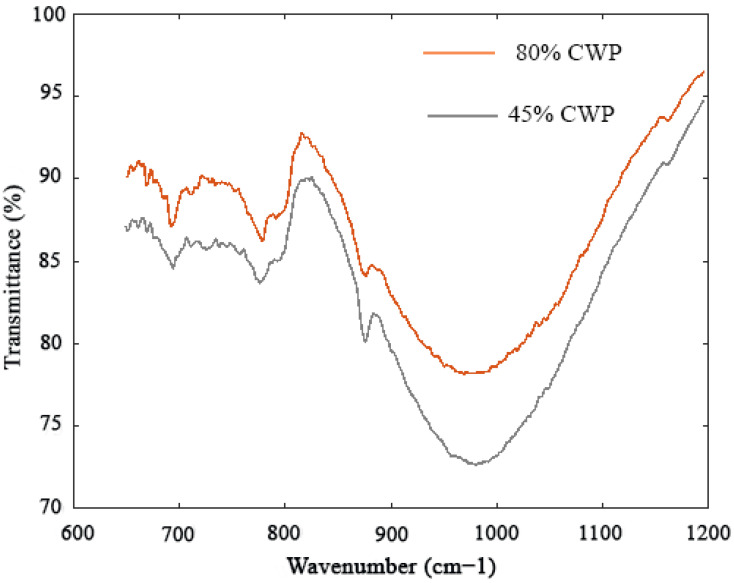
FTIR results of the alkali-activated binder prepared with high amount of CWP.

In addition, Figure 7 shows the FTIR analysis results of the SCAACs prepared with various levels of CWP. The strength results indicated that the inclusion of CWP in the alkali-activated matrix reduced the specimen strength. This is because the amounts of dissolved CaO and Al_2_O_3_ decrease as the concentration of GBFS is minimized. Similarly, it was found that the lack of dissolved Al_2_O_3_ leads to a diminished extent of silicate polymerization [43]. It is well known that the FTIR test aims to analyse the bonding vibrations within the concrete specimen, which reflect the compressive strength, specifically through the occurrence of an FTIR spectral band of Si-O-Al. It can be seen that the band frequency increased when the concentration of CWP increased from 45% to 80%. In addition, the increase in the FTIR vibration frequency was due to the decrease in the Al_2_O_3_ level in the SCAAC mixes.

### 3.3. Effect of CWP Incorporation on the Durability of Acid-Treated AAC

**Figure 8:**
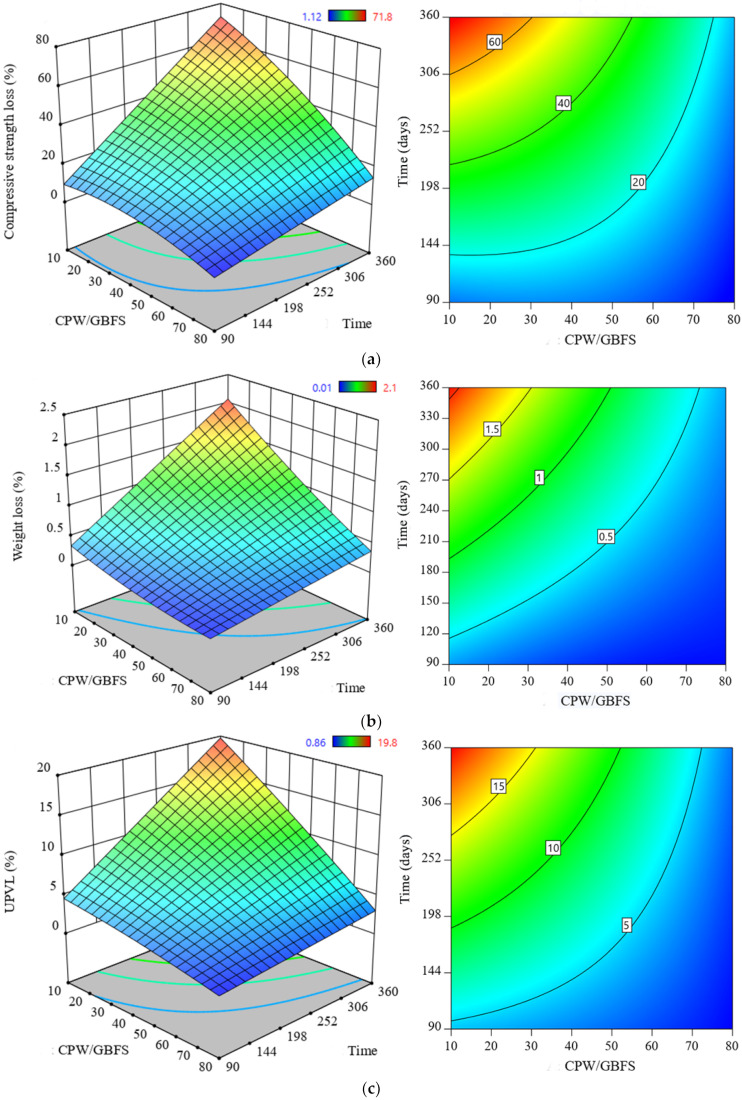
Durability of SCAAC exposed to acid: (**a**) strength loss, (**b**) weight loss, and (**c**) UPVL.

The durability performance of the proposed SCAAC in an aggressive environment was evaluated, and a sulphuric acid environment was considered for this purpose. The losses in strength, weight, and UPV were measured after 3, 6, and 12 months from the immersion date in the 10% H_2_SO_4_ solution. The effects of the GBFS replacement with the CWP on the durability of the SCAAC in terms of strength, weight, and UPV losses were recorded, and the results are presented in Figure 8. For the strength loss, it was observed that the inclusion of CWP resulted in an enhanced durability in the prepared specimen and a reduced strength loss percentage from 74% to 13.3% with an increase in the CWP content from 0% to 80% for the specimen exposed to acid solution for 12 months (Figure 8a). Likewise, the weight loss decreased gradually with increasing replacement level of the GBFS by the CWP in the binder matrix, as shown in Figure 8b. For example, the weight loss percentage dropped from 2.2% to 0.39% when the CWP content increased from 0% to 80%. This is because the formation of excessive undesirable gypsum in the control mix led to softening of the binder, and therefore, the surface of the concrete significantly deteriorated. In particular, the paste was separated from the aggregate and thus reduced the cross-section of the concrete samples. In contrast, the AAC incorporating a high amount of CWP resists binder deterioration and protects the concrete surface owing to the limited amount of calcium ions. This is also in line with previous studies [44,45]. On the other hand, the UPV test was conducted on the specimens exposed to acid solution for 12 months to measure the internal deterioration of the mixes. Figure 8c presents the loss in UPV percentage results, which indicated a loss percentage drop from 20.2% to 3.1% with an increase in the replacement level of the GBFS by the WCP from 0% to 80%.

**Figure 9:**
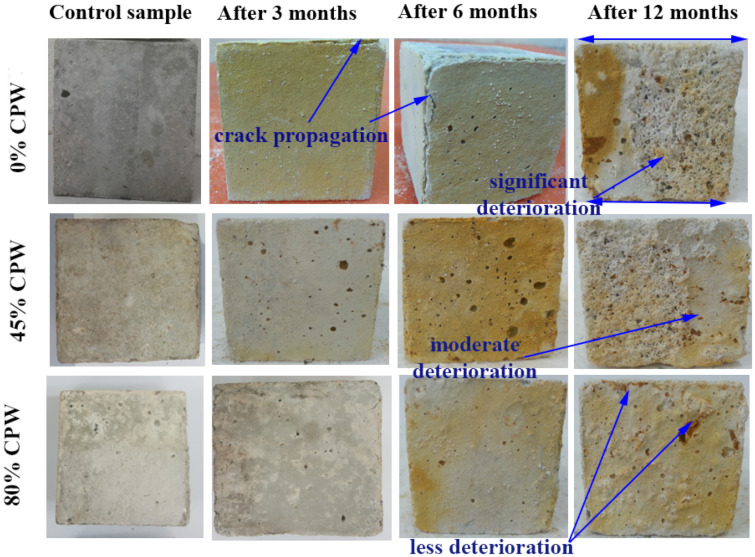
Surface texture of the proposed SCAAC containing 0, 50, and 70% CWP exposed to 10% H_2_SO4 after different immersion periods.

Moreover, the formation of cracks and surface deterioration decreased as the CWP content increased, as shown in Figure 9. Based on the visual examination, the CWP/GBFS-based AAC appeared to be in good condition compared to that of the control mix. The worsening of the GBFS-based ACC prepared with 45% and 80% CWP was less than that of the OPC. The cross section of the GFBS-based ACC containing 80% after 3, 6, and 12 months was almost the same. On the other hand, with the increase in exposure time, a significant reduction in the cross-section of the control mix was observed. In particular, crack formation occurred after three months and extended to six months. Then, a part of the concrete was removed, resulting in a reduction in the cross-section of the OPC owing to the softening of the paste around the aggregates. In addition, this was due to the high amount of calcium in the control specimen without ceramic incorporation, which resulted in a high amount of gypsum product. A concrete specimen with a high CWP content exhibited a high resistance to sulphuric acid attack. In other words, the enhancement in the durability of the proposed concrete to an acidic environment is attributed to the reduction in CaO and Ca(OH)_2_ levels accompanied by an increase in CWP content. It is well known that the high availability of SO_4_^−2^ in sulphuric acid can react with Ca(OH)_2_ in concrete to form gypsum (CaSO_4_.2H_2_O). This product exhibited several undesirable results, in which an expansion could be generated in the concrete matrix to form further cracks. Consequently, such negative results could prompt and increase the deterioration of alkali-activated concrete [26,46]. This is also in good agreement with Allahverdi and Skvara [47], who utilized 50% GBFS in sodium-silicate-activated specimens for exposure to an acid solution. The results showed that the presence of Na^+^ and Ca^++^ negatively affected the acid resistance of the concrete. In particular, the degradation of the gel product was accelerated owing to the exchange reaction between the OH ions with Ca^++^ and Na^+^. Moreover, the composition and structure of the Al_2_O_3_-SiO_2_ network underwent changes due to de-alumination [48].

## 4. Conclusions

Self-compacting alkali-activated concrete has been widely applied and experimentally evaluated using different binders, such as POFA, FA, and GBFS. However, the utilization of CWP as a binder in GBFS-based SCAACs is still limited. In the present study, an optimization method was considered to assess and obtain the optimal CWP content to satisfy both the strength and durability aspects. Based on the visual examination, predicted, and actual results, it can be concluded that:

- As a benefit of the RSM model, the number of experiments was found to be small enough (13) to develop a predictive equation.
- RSM proved its ability to assess the behavior of SCAAC incorporating CWP, in which the appropriate correlation between the actual data and the predicted data was achieved. In particular, the *R*, *R*^2^, and adjusted *R*^2^ values were higher than 0.95.
- The significance of the developed models was also proven by the high F-value and *p*-value of less than 0.0001. In addition, the proposed models can accurately predict the behavior of the SCAAC with minimum errors (RMSE < 1.337).
- The high replacement of GBFS by CWP prevented the deterioration of the concrete surface owing to the limited amount of calcium ions.
- The optimum replacement percentage of GBFS by CWP was 31%, in which a reasonable decrease in the compressive strength (16%) was obtained in addition to the minimized strength loss of the SCACC when exposed to an acid attack of only 59.17% (compared to 74.2% for the control specimen).
- The strength and weight loss of the SCAAC significantly decreased with the increase in the amount of CWP, specifically, 45% and 80%, respectively.

Therefore, it could be concluded that CWP could serve as an excellent strategic material to address serious environmental issues and enhance the durability of SCAAC-exposed acid attacks.

## Figures and Tables

**Table 1:** The suggested design array of experimental work based on FC-CCD.

Run No.	Coded Value		Real Value			FC-CCD Division
			Model 1 and 2 CWP/GBFS (%)	Model 1 Time (Days)	Model 2 Time (Days)	
1	−1	−1	10	3	90	Factorial points (2n)
2	1	−1	80	3	90	
3	−1	1	10	56	360	
4	1	1	80	56	360	
5	1	0	10	29.5	225	Axial points (2n)
6	−1	0	80	29.5	225	
7	0	−1	45	3	90	
8	0	1	45	56	360	
9	0	0	45	29.5	225	Centre points
10	0	0	45	29.5	225	
11	0	0	45	29.5	225	
12	0	0	45	29.5	225	
13	0	0	45	29.5	225	

**Table 2:** Predictive equations of SCAAC properties.

Model	Item (MPa)	Predicted Equations and Related Statistics Indicators					
**Model 1**	**Compressive strength**	R = 0.995	R^2^ = 0.992	$R_{adj}^{2}=$ 0.987	$R_{predicted}^{2}=$ 0.931	Adeq. Precision 47.938	RMSE 1.337
		$CS=47.11-18.57d_{0}+8.84d_{1}-3.05d_{0}d_{1}-1.31d_{0}^{2}-3.29d_{1}^{2}$					
	**Tensile strength**	R = 0.998	R^2^ = 0.997	$R_{adj}^{2}=$ 0.995	$R_{predicted}^{2}=$ 0.971	Adeq. Precision 74.47	RMSE 0.0631
		$TS=4.62-1.36d_{0}+0.55d_{1}-0.125d_{0}d_{1}-0.26d_{0}^{2}-0.43d_{1}^{2}$					
	**Flexural strength**	R = 0.996	R^2^ = 0.992	$R_{adj}^{2}=$ 0.987	$R_{predicted}^{2}=$ 0.931	Adeq. Precision 49.11	RMSE 0.0349
		$FS=1.49-0.39d_{0}+0.29d_{1}-0.05d_{0}d_{1}+0.09d_{0}^{2}-0.21d_{1}^{2}$					
**Model 2**	**Strength loss**	R = 0.998	R^2^ = 0.997	$R_{adj}^{2}=$ 0.995	$R_{predicted}^{2}=$ 0.974	Adeq. Precision 83.192	RMSE 1.022
		$SL=29.26-16.95d_{0}+19.38d_{1}-12.30d_{0}d_{1}-75.1d_{0}^{2}-0.0008d_{1}^{2}$					
	**weight loss**	R = 0.9988	R^2^ = 0.9978	$R_{adj}^{2}=$ 0.996	$R_{predicted}^{2}=$ 0.977	Adeq. Precision 88.7	RMSE 0.0269
		$WL=0.625-0.5d_{0}+0.51d_{1}-0.35d_{0}d_{1}+0.07d_{0}^{2}-0.0002d_{1}^{2}$					
	**UPVL**	R = 0.9999	R^2^ = 0.9999	$R_{adj}^{2}=$ 0.9998	$R_{predicted}^{2}=$ 0.9987	Adeq. Precision 362.72	RMSE 0.0625
		$UPVL=7.39-5.09d_{0}+4.33d_{1}-3.26d_{0}d_{1}-0.325d_{0}^{2}$					

**Table 3:** Validation of the proposed models using ANOVA.

Model	Type	ANOVA	Term					
			Model	d_0_	d_1_	d_0_ d_1_	d_0_^2^	d_1_^2^
Model 1	CS	*p*-value	<0.0001	<0.0001	<0.0001	0.0085	0.2357	0.0141
		F-value	185.37	730.08	165.57	13.13	1.68	10.54
		Sig.	Y	-	-	-	-	-
	TS	*p*-value	<0.0001	<0.0001	<0.0001	0.0131	0.0007	<0.0001
		F-value	491.75	1930.24	325.15	10.90	32.73	90.37
		Sig.	Y	-	-	-	-	-
	FS	*p*-value	<0.0001	<0.0001	<0.0001	0.0335	0.0089	<0.0001
		F-value	186.05	542.72	308.71	6.96	12.86	71.70
		Sig.	Y	-	-	-	-	-
Model 2	SL	*p*-value	<0.0001	<0.0001	<0.0001	<0.0001	0.0003	0.9992
		F-value	564.82	1042.85	1363.63	366.61	43.58	1.1E-06
		Sig.	Y	-	-	-	-	-
	WL	*p*-value	<0.0001	<0.0001	<0.0001	<0.0001	0.0067	0.9941
		F-value	635.05	1324.13	1399.85	434.40	14.44	0.0001
		Sig.	Y	-	-	-	-	-
	UPVL	*p*-value	<0.0001	<0.0001	<0.0001	<0.0001	0.0002	1.0
		F-value	10641.2	26589.5	19250.2	7308.08	50.0	0.0
		Sig.	Y	-	-	-	-	-

## Data Availability

The data presented in this study are available on request from the corresponding author. The data are not publicly available due to data privacy.
